# Prevalence of undernutrition and potential risk factors among children under 5 years of age in Amhara Region, Ethiopia: evidence from 2016 Ethiopian Demographic and Health Survey

**DOI:** 10.1017/jns.2021.17

**Published:** 2021-04-08

**Authors:** Damitie Kebede, Yidnekachew Merkeb, Eyerusalem Worku, Hayat Aragaw

**Affiliations:** 1 College of Agriculture and Environmental Sciences, Bahir Dar University, P.O. Box 5501, Bahir Dar, Ethiopia; 2 Department of Applied Human Nutrition, Faculty of Chemical and Food Engineering, Bahir Dar Institute of Technology, Bahir Dar University, P.O. Box 79, Bahir Dar, Ethiopia; 3 Institute of Disaster Risk Management and Food Security Studies, Bahir Dar University, P.O. Box 5501, Bahir Dar, Ethiopia

**Keywords:** Ethiopia, Stunting, Underweight, Wasting, Young children, AOR, adjusted odds ratio, BMI, body mass index, COR, crude odds ratio, CSA, Central Statistical Agency, DHS, Demographic and Health Surveys, EDHS, Ethiopian Demographic and Health Survey, SNNPR, South Nations, Nationalities, and Peoples’ Region, SPSS, Statistical Package for Social Science

## Abstract

More than one-third of deaths during the first 5 years of life are attributed to undernutrition, which are mostly preventable through economic development and public health measures. The present study aimed to explore the potential risk factors of undernutrition among children under 5 years of age in Amhara Region, Ethiopia. Data from the 2016 Ethiopian Demographic and Health Survey (EDHS) were used. A total of 974 children under 5 years of age were involved. A multivariable binary logistic regression analysis was used at a 5 % level of significance to determine the individual- and community-level factors associated with childhood undernutrition. The prevalence of stunting, wasting and underweight was 46⋅3, 9⋅8 and 28⋅4 %, respectively. About 23⋅1 % of children were both stunting and underweight, 7⋅3 % were both underweight and wasting and 4⋅5 % of children had all three conditions. Among the factors considered in the present study, the age of a child in months, birth weight, mother educational level, sex of household head, sources of drinking water and the type of toilet facility were significantly associated with undernutrition in the Amhara Region. Undernutrition among under-five children was one of the public health problems in the Amhara Region. The potential risk factors should be considered to develop strategies for reducing undernutrition in the Amhara Region. Finally, improving the living standards of the children is important to get better health care, to enhance the child's nutritional status and to reduce child mortality.

## Background

Undernutrition among children continues to be a major public health problem throughout the world. Globally, one in every three under-five children is undernourished. In 2018, about 155 million children under the age of 5 years were stunted and 52 million wasted worldwide. Asia and Africa contributed 56 and 38 % of stunting and 69 and 27⋅2 % of wasting, respectively, of the global undernutrition burden^(1)^. Wasting is a measure of acute undernutrition, and it may result from inadequate food intake or from a recent episode of illness that caused weight loss. Stunting is a sign of chronic undernutrition that reflects a failure to receive adequate nutrition over a long period and that can also be affected by recurrent and chronic illness^(2)^.

In the Amhara Region, the prevalence of stunting declined from 56⋅6 % in 2005 to 52 % in 2011; the prevalence of wasting decreased from 14⋅2 % in 2005 to 9⋅9 % in 2011 and the prevalence of being underweight sharply decreased from 48⋅9 % in 2005 to 33⋅4 % in 2011. The prevalence of stunting declined from 58 % in 2000 to 44 % in 2011 in Ethiopia. The prevalence of wasting changed from 12 % in 2000 to 10 % in 2011. The prevalence of being underweight steadily decreased from 41 % in 2000 to 29 % in 2011^(3)^. Although the prevalence of undernutrition in the Amhara Region has declined, still it is a major public health problem^(4)^.

Undernutrition in children occurs due to the interplay of several factors, which include variables related to the maternal age, maternal education, poor feeding practice, maternal nutritional status, parity and multiple births, sex of the child, illness, birth interval and immunisation status, poor wealth status, large families, water and sanitation, place of residence and other factors relating to health services utilisation^(5)^.

Although a few studies have been carried out on the identification of factors that are associated with children under 5 years old of undernutrition in the Amhara Region, none of them uses the nationally representative data for the Amhara Region. The effort made in decreasing under-five children's undernutrition in the region is still high, and more effort is needed to improve the barriers for further reduction. More research studies are, therefore, required to inform policymakers to implement appropriate intervention programmes. To address these gaps, an all-inclusive cross-sectional analysis of the recent 2016 Ethiopian Demographic Health Survey (EDHS) was done, to explore the potential risk factors of undernutrition among under-five children in Amhara Region, Ethiopia.

## Methods

### Description of the study design and area

A cross-sectional study design was used for the present study. The present study was conducted in the Amhara regional state which is located in the northwestern and northcentral parts of Ethiopia. According to the 2007 Census, the state's population birth weight was 17 214 056 of which 8 636 1875 were males. The urban residents of the region were 2 112 220 and its rural residents were 15 101 836^(6)^.

### Source of data

The data for the present study was extracted from the EDHS 2016. The Central Statistics Agency (CSA), the Ministry of Health (MOH) and the Ethiopian Public Health Institute together surveyed from 18 January 2016 to 27 June 2016, where the United States Agency for International Development (USAID) funded the project. The authors have got permission from the ICF-DHS program to use the EDHS data and access it through https://www.dhsprogram.com/data/dataset_admin/login_main.cfm. The 2016 EDHS used a two-stage stratified sampling to select households. In the first stage, there were 645 enumeration areas (202 in urban and 443 in rural areas). About 974 (501 males and 473 females) under-five children from the Amhara Region were considered for the present study. The present study considered live children age 0–59 months with anthropometry data in the analysis of determinants of nutritional status among children under age 5 in the Amhara Region. Missing values in the 2016 EDHS dataset were not included in the analyses.

### Study variables

The dependent variables for the present study are the undernutrition status of under5-year children (stunting, underweight and wasting). A child whose height for age *Z*-score is below minus two standard deviations (−2sd) from the median of the reference population is considered as stunting. If the weight for age *Z*-score is below minus two standard deviations (−2sd) from the median of the reference population, then the child is underweight. Children whose weight for height *Z*-score is below minus two standard deviations (−2sd) from the median of the reference population are considered as wasting. Malnutrition indicators were defined using the WHO child growth standards^(2)^.

The determinants of stunting, wasting and underweight in the present study were selected from the available similar studies on the subject^(7–11)^. The risk factors associated included socio-demographic, maternal and child factors. Socio-demographic–maternal factors selected were the types of residence, household wealth index, mothers’ educational level, mothers’ body mass index (BMI), religion, the type of toilet facility, sex of household head and sources of drinking water. Child-level factors were the sex of the child, child age, the type of birth, the number of living children and birth weight.

### Statistical data analysis

The data were extracted, edited and analysed by using SPSS version 23 for Windows. Descriptive statistics such as frequencies and proportions were used to summarise the distribution of selected background characteristics of the sample. Bivariate logistic regression was performed to analyse the crude odds ratio (COR), and a variable with a *P*-value of less than 0⋅25 was transported into multivariable binary logistic regression analyses to analyse the adjusted odds ratio (AOR) and to identify the determinants of undernutrition of children under 5 years old. The dependent variables for bivariate and multivariable logistic regression analyses were stunting, wasting and underweight. Finally, variables with *P*-values <0⋅05 in the multivariable logistic regression model were taken as statistically significant.

## Results

Our population included 974 children under the age of 5 years from the Amhara Region who were considered in this research. The prevalence of stunting, underweight and wasting in the Amhara Region was 46⋅3, 28⋅4 and 9⋅8 %, respectively. About 23⋅1 % of children were both stunted and underweight, 7⋅3 % were both underweight and wasting and 4⋅5 % of children had all three conditions.

Of that 23⋅3 % were >4 kg birth weight and only 2⋅9 % were multiple birth types. About 74⋅9 % of interviewed mothers were illiterate and only 2⋅1 % of them attended diploma and above. About 42⋅6 % of children were found between 0 and 24 months, and more than half (51⋅3 %) were males. Only 9⋅1 % of the respondents were from urban areas and 30⋅2 % were in the rich wealth index. About 19⋅8 % of children's mothers were underweight.

### Determinants of stunting

Among the factors considered in the present study, the age of child in months, birth weight, mother educational level, sex of household head and sources of drinking water were associated with stunting. The log odds of stunting was higher among children in the age group of 25–47 months (AOR 1⋅57; 95 % CI 1⋅05, 2⋅35) and 48–59 months (AOR 1⋅06; 95 % CI 0⋅80, 1⋅49), respectively, as compared with the age group of 0–24 months. Compared with children >4 kg birth weight at birth, the odds of stunting among children in the 2⋅5–4 kg birth weight were 0⋅01 times lower. The odds of stunting among children in the <2⋅5 kg birth weight were 1⋅60 times higher compared with children >4 kg birth weight at birth.

The risk of stunting among children whose mothers attended primary education was 1⋅07 (AOR 1⋅07; 95 % CI 0⋅76, 1⋅50) times more compared with children whose mothers did not attend education. The risk of being stunted among children whose mothers attended secondary education was 0⋅70 times less compared with children whose mothers were illiterate. The risk of stunting among children whose father household head was 0⋅49 times less compared with children whose mother household headed. Children from households that used unimproved drinking water were 1⋅47 (AOR 1⋅47; 95 % CI 1⋅11, 1⋅95) times more likely to be at risk of stunting than children from households that used improved water (Table 1).

**Table 1.** Bivariate and multivariable logistic regression of risk factors associated with stunting on under-five children in Amhara Region, Ethiopia, EDHS 2016 (Odds ratios and 95% confidence intervals)

Variables	Stunting		COR (95 % CI)	AOR (95 % CI)
	Yes	No		
Age of child in months				
0–24	169 (43⋅7 %)	218 (56⋅3 %)	1	1
25–47	149 (45⋅8 %)	176 (54⋅2 %)	1⋅09 (0⋅81, 1⋅47)	1⋅57 (1⋅05, 2⋅35)*
48–59	102 (51⋅5 %)	96 (48⋅5 %)	1⋅37 (0⋅97, 1⋅93)	1⋅06 (0⋅80, 1⋅49)
Sex of child				
Male	218 (43⋅6 %)	282 (56⋅4 %)	1	1
Female	232 (48⋅9 %)	242 (51⋅1 %)	1⋅24 (0⋅96, 1⋅60)	1⋅27 (0⋅97, 1⋅67)
Place of residence				
Urban	39 (43⋅8 %)	50 (56⋅2 %)	1	1
Rural	411 (46⋅4 %)	474 (53⋅6 %)	1⋅11 (0⋅72, 1⋅72)	0⋅53 (0⋅26, 1⋅06)
Religion				
Orthodox	380 (46⋅3 %)	441 (53⋅7 %)	1	1
Muslin	70 (46⋅1 %)	82 (53⋅9 %)	0⋅99 (0⋅70, 1⋅40)	1⋅07 (0⋅69, 1⋅66)
Type of birth				
Single birth	438 (46⋅3 %)	508 (53⋅7 %)	1	1
Multiple birth	12 (42⋅9 %)	16 (57⋅1 %)	0⋅87 (0⋅41, 1⋅86)	1⋅18 (0⋅41, 3⋅39)
Mothers’ BMI				
Overweight	15 (41⋅7 %)	21 (58⋅3 %)	1	1
Normal weight	351 (47⋅6 %)	387 (52⋅4 %)	1⋅01 (0⋅49, 2⋅06)	0⋅95 (0⋅37, 2⋅43)
Underweight	81 (41⋅8 %)	113 (58⋅2 %)	1⋅40 (0⋅30, 6⋅51)	0⋅78 (0⋅10, 6⋅21)
Birth weight				
>4 kg	99 (43⋅6 %)	128 (56⋅4 %)	1	1
2⋅5–4 kg	176 (42⋅1 %)	242 (57⋅9 %)	0⋅94 (0⋅68, 1⋅30)	0⋅99 (0⋅70, 1⋅42)
<2⋅5 kg	175 (53⋅2 %)	154 (46⋅8 %)	1⋅47 (1⋅05, 2⋅07)	1⋅60 (1⋅11, 2⋅31)**
Mother educational level				
Illiterate	343 (46⋅9 %)	388 (53⋅1 %)	1	1
Primary	87 (47⋅3 %)	97 (52⋅7 %)	1⋅02 (0⋅72, 1⋅40)	1⋅07 (0⋅76, 1⋅50)
Secondary	7 (18⋅4 %)	31 (81⋅6 %)	0⋅26 (0⋅11, 0⋅59)	0⋅30 (0⋅13, 0⋅70)*
Diploma and above	7 (18⋅4 %)	31 (81⋅6 %)	1⋅84 (0⋅75, 4⋅49)	2⋅37 (0⋅87, 6⋅52)
Type of toilet facility				
Improved	236 (48⋅0 %)	256 (52⋅0 %)	1	1
Unimproved	215 (44⋅6 %)	267 (55⋅4 %)	1⋅15 (0⋅89, 1⋅47)	1⋅30 (0⋅96, 1⋅77)
Sex of household head				
Female	36 (40⋅4 %)	53 (59⋅6 %)	1	1
Male	414 (46⋅8 %)	471 (53⋅2 %)	1⋅29 (0⋅83, 2⋅02)	0⋅51 (0⋅28, 0⋅91)*
Household wealth index combined				
Poor	210 (46⋅7 %)	240 (53⋅3 %)	1	1
Medium	109 (47⋅4 %)	121 (52⋅6 %)	1⋅03 (0⋅75, 1⋅42)	1⋅04 (0⋅71, 1⋅52)
Rich	131 (44⋅6 %)	163 (55⋅4 %)	0⋅92 (0⋅68, 1⋅23)	0⋅77 (0⋅52, 1⋅14)
Number of living children				
1–2	160 (45⋅8 %)	189 (54⋅2 %)	1	1
3–4	80 (46⋅8 %)	91 (53⋅2 %)	0⋅99 (0⋅75, 1⋅31)	1⋅08 (0⋅77, 1⋅52)
>4	207 (46⋅1 %)	242 (53⋅9 %)	1⋅03 (0⋅72, 1⋅46)	1⋅26 (0⋅82, 1⋅94)
Sources of drinking water				
Improved	178 (50⋅9 %)	172 (49⋅1 %)	1	1
Unimproved	273 (43⋅8 %)	351 (56⋅2 %)	0⋅75 (0⋅58, 0⋅98)	1⋅47 (1⋅11, 1⋅95)**

AOR, adjusted odds ratio; COR, crude odds ratio; CI, confidence interval; 1, reference.

* *P*-value <0⋅05 is considered significant.

** *P*-value <0⋅01 is considered significant.

### Determinants of underweight

The birth weight was associated with underweight (*P* < 0⋅05). The risk of being underweight was 0⋅22 times less likely among children that were aged 25–47 months than those aged 0–24 months. The risk of being underweight was 1⋅36 times more likely among children that were aged 48–59 months than those aged 0–24 months. The risk of being underweight for children whose mother attended primary and secondary education were 0⋅23 and 0⋅35 times lower than children whose mothers who did not attend formal education, respectively. Children from a household with rich economic status were 0⋅09 times less likely to be underweighted compared with children living in a household with poor household economic status. Children from rural areas were 1⋅16 times more likely to be underweight compared with children from urban areas. Children who were born with <2⋅5 kg birth weight were 1⋅80 times more likely to be underweight than children born >4 kg birth weight (AOR 1⋅80; 95 % CI 0⋅89, 3⋅66) and children who had born with 2⋅5–4 kg birth weight were 1⋅56 times more likely to be underweighted than children born >4 kg birth weight (AOR 1⋅56; 95 % CI 1⋅05, 2⋅33) (Table 2).

**Table 2.** Bivariate and multivariable logistic regression of risk factors associated with underweight under-five children in Amhara Region, Ethiopia, EDHS 2016 (Odds ratios and 95 % confidence intervals)

Variables	Underweight		COR (95 % CI)	AOR (95 % CI)
	Yes	No		
Age of child in months				
0–24	104 (26⋅9 %)	283 (73⋅1 %)	1	1
25–47	86 (26⋅5 %)	239 (73⋅5 %)	0⋅98 (0⋅70, 1⋅37)	0⋅78 (0⋅53, 1⋅16)
48–59	63 (31⋅8 %)	135 (68⋅2 %)	1⋅27 (0⋅87, 1⋅85)	1⋅36 (0⋅89, 2⋅09)
Sex of child				
Male	134 (26⋅8 %)	366 (73⋅2 %)	1	1
Female	142 (30⋅0 %)	332 (70⋅0 %)	1⋅17 (0⋅88, 1⋅54)	1⋅06 (0⋅76, 1⋅49)
Place of residence				
Urban	15 (16⋅9 %)	74 (83⋅1 %)	1	1
Rural	261 (29⋅5 %)	624 (70⋅5 %)	2⋅06 (1⋅16, 3⋅66)	1⋅16 (0⋅50, 2⋅69)
Religion				
Orthodox	228 (27⋅8 %)	593 (72⋅2 %)	1	1
Muslin	48 (31⋅6 %)	104 (68⋅4 %)	1⋅20 (0⋅83, 1⋅75)	1⋅60 (1⋅01, 2⋅54)
Type of birth				
Single birth	268 (28⋅3 %)	678 (71⋅7 %)	1	1
Multiple birth	8 (28⋅6 %)	20 (71⋅4 %)	1⋅01 (0⋅44, 2⋅32)	1⋅32 (0⋅43, 4⋅03)
Mothers’ BMI				
Overweight	8 (22⋅2 %)	28 (77⋅8 %)	1	1
Normal weight	209 (28⋅3 %)	529 (71⋅7 %)	1⋅46 (0⋅63, 3⋅39)	2⋅28 (0⋅63, 8⋅27)
Underweight	57 (29⋅4 %)	137 (70⋅6 %)	1⋅17 (0⋅20, 6⋅94)	0⋅88 (0⋅07, 11⋅51)
Birth weight				
>4 kg	61 (26⋅9 %)	166 (73⋅1 %)	1	1
2⋅5–4 kg	101 (24⋅2 %)	317 (75⋅8 %)	0⋅87 (0⋅60, 1⋅25)	1⋅56 (1⋅05, 2⋅33)*
<2⋅5 kg	114 (34⋅7 %)	215 (65⋅3 %)	1⋅44 (0⋅99, 2⋅09)	1⋅80 (0⋅89, 3⋅66)
Mother educational level				
Illiterate	217 (29⋅7 %)	514 (70⋅3 %)	1	1
Primary	47 (25⋅5 %)	137 (74⋅5 %)	0⋅82 (0⋅56, 1⋅17)	0⋅89 (0⋅58, 1⋅38)
Secondary	5 (13⋅2 %)	33 (86⋅8 %)	0⋅36 (0⋅14, 0⋅93)	0⋅25 (0⋅05, 1⋅14)
Diploma and above	7 (33⋅3 %)	14 (66⋅7 %)	1⋅18 (0⋅47, 2⋅96)	1⋅92 (0⋅50, 7⋅34)
Type of toilet facility				
Improved	130 (26⋅4 %)	362 (73⋅6 %)	1	1
Unimproved	146 (30⋅3 %)	336 (69⋅7 %)	0⋅83 (0⋅62, 1⋅09)	0⋅80 (0⋅57, 1⋅12)
Sex of household head				
Female	25 (28⋅1 %)	64 (71⋅9 %)	1	1
Male	251 (28⋅4 %)	634 (71⋅6 %)	0⋅99 (0⋅61, 1⋅60)	0⋅53 (0⋅27, 1⋅05)
Household wealth index combined				
Poor	133 (29⋅6 %)	317 (70⋅4 %)	1	1
Medium	67 (29⋅1 %)	163 (70⋅9 %)	0⋅98 (0⋅69, 1⋅39)	1⋅07 (0⋅71, 1⋅61)
Rich	76 (25⋅9 %)	218 (74⋅1 %)	0⋅83 (0⋅60, 1⋅16)	0⋅91 (0⋅59, 1⋅40)
Number of living children				
1–2	85 (24⋅4 %)	264 (75⋅6 %)	1	1
3–4	54 (31⋅6 %)	117 (68⋅4 %)	0⋅75 (0⋅55, 1⋅03)	0⋅86 (0⋅59, 1⋅26)
>4	135 (30⋅1 %)	314 (69⋅9 %)	0⋅1⋅07 (0⋅73, 3⋅90)	1⋅25 (0⋅79, 1⋅97)
Sources of drinking water				
Improved	113 (32⋅3 %)	237 (67⋅7 %)	1	1
Unimproved	163 (26⋅1 %)	461 (73⋅9 %)	1⋅35 (1⋅01, 1⋅80)	1⋅20 (0⋅85, 1⋅69)

AOR, adjusted odds ratio; COR, crude odds ratio; CI, confidence interval; 1, reference.

* *P*-value <0⋅05 is considered significant.

### Determinants of wasting

Results of the multivariable binary logistic regression model showed that the type of toilet facility and sex of household head were significantly associated with wasting. Children living in a household with improved toilet type were 0⋅48 less likely to be wasting compared with children living in a household with unimproved toilet type. Children from a male household head were 1⋅99 times higher compared with children from a female household head. Children from the rich household were 0⋅32 times less likely to be wasted compared with children living in a household with poor household economic status.

The risk of wasting was 1⋅08 and 1⋅52 times higher among children of 25–47 and 48–59 months than those of 0–24 months, respectively. The odds of wasting children from rural areas were 1⋅18 times higher compared with children from urban areas. The odds of wasting were 0⋅04 times lower among female children than male children. The risk of being wasted was 2⋅96 times higher among children who were born from illiterate mothers than those born from diploma and above holder mothers. Children who lived in household members >4 were 1⋅17 times more likely to be wasted than children who lived in household members of 1–2 (AOR 1⋅17; 95 % CI 0⋅59, 2⋅33) (Table 3).

**Table 3.** Bivariate and multivariable logistic regression of risk factors associated with wasting on under-five children in Amhara Region, Ethiopia, EDHS 2016 (Odds ratios and 95 % confidence intervals)

Variables	Wasting		COR (95 % CI)	AOR (95 % CI)
	Yes	No		
Age of child in months				
0–24	33 (8⋅5 %)	354 (91⋅5 %)	1	1
25–47	30 (9⋅2 %)	295 (90⋅8 %)	1⋅09 (0⋅65, 1⋅83)	1⋅08 (0⋅64, 1⋅82)
48–59	25 (12⋅6 %)	173 (87⋅4 %)	1⋅55 (0⋅89, 2⋅69)	1⋅52 (0⋅87, 2⋅64)
Sex of child				
Male	50 (10⋅0 %)	450 (90⋅0 %)	1	1
Female	46 (9⋅7 %)	428 (90⋅3 %)	0⋅97 (0⋅63, 1⋅48)	0⋅96 (0⋅58, 1⋅58)
Place of residence				
Urban	7 (7⋅9 %)	82 (92⋅1 %)	1	1
Rural	89 (10⋅1 %)	796 (89⋅9 %)	1⋅31 (0⋅59, 2⋅92)	1⋅18 (0⋅36, 3⋅88)
Religion				
Orthodox	79 (9⋅6 %)	742 (90⋅4 %)	1	1
Muslin	17 (11⋅2 %)	135 (88⋅8 %)	1⋅18 (0⋅68, 2⋅06)	1⋅22 (0⋅62, 2⋅37)
Type of birth				
Single birth	94 (9⋅9 %)	852 (90⋅1 %)	1	1
Multiple birth	2 (7⋅1 %)	26 (92⋅9 %)	0⋅68 (0⋅16, 2⋅98)	0⋅53 (0⋅06, 4⋅32)
Mothers’ BMI				
Overweight	5 (13⋅9 %)	31 (86⋅1 %)	1	1
Normal weight	71 (9⋅6 %)	667 (90⋅4 %)	0⋅67 (0⋅23, 1⋅94)	2⋅50 (0⋅32, 19⋅44)
Underweight	19 (9⋅8 %)	175 (90⋅2 %)	0⋅89 (0⋅09, 8⋅82)	2⋅73 (0⋅34, 22⋅08)
Birth weight				
>4 kg	22 (9⋅7 %)	205 (90⋅3 %)	1	1
2⋅5–4 kg	46 (11⋅0 %)	372 (89⋅0 %)	1⋅15 (0⋅67, 1⋅97)	1⋅36 (0⋅71, 2⋅61)
<2⋅5 kg	28 (8⋅5 %)	301 (91⋅5 %)	0⋅87 (0⋅48, 1⋅56)	0⋅87 (0⋅42, 1⋅77)
Mother educational level				
Illiterate	72 (9⋅8 %)	659 (90⋅2 %)	1	1
Primary	18 (9⋅8 %)	166 (90⋅2 %)	0⋅99 (0⋅58, 1⋅71)	1⋅32 (0⋅71, 2⋅46)
Secondary	4 (10⋅5 %)	34 (89⋅5 %)	1⋅08 (0⋅37, 3⋅12)	1⋅63 (0⋅39, 6⋅73)
Diploma and above	2 (9⋅5 %)	19 (90⋅5 %)	0⋅96 (0⋅22, 4⋅22)	2⋅96 (0⋅46, 19⋅07)
Type of toilet facility				
Improved	39 (7⋅9 %)	453 (92⋅1 %)	1	1
Unimproved	57 (11⋅8 %)	425 (88⋅2 %)	0⋅64 (0⋅42, 0⋅99)	0⋅52 (0⋅31, 0⋅87)**
Sex of household head				
Female	14 (15⋅7 %)	75 (84⋅3 %)	1	1
Male	82 (9⋅3 %)	803 (90⋅7 %)	1⋅83 (0⋅99, 3⋅39)	1⋅99 (1⋅06, 3⋅73)*
Household wealth index combined				
Poor	46 (10⋅2 %)	404 (89⋅8 %)	1	1
Medium	29 (12⋅6 %)	201 (87⋅4 %)	1⋅27 (0⋅77, 2⋅08)	1⋅12 (0⋅66, 1⋅90)
Rich	21 (7⋅1 %)	273 (92⋅9 %)	0⋅68 (0⋅39, 1⋅16)	0⋅68 (0⋅38, 1⋅19)
Number of living children				
1–2	30 (8⋅6 %)	319 (91⋅4 %)	1	1
3–4	17 (9⋅9 %)	154 (90⋅1 %)	0⋅77 (0⋅48, 1⋅24)	1⋅04 (0⋅59, 1⋅82)
>4	49 (10⋅9 %)	400 (89⋅1 %)	0⋅90 (0⋅53, 1⋅61)	1⋅17 (0⋅59, 2⋅33)
Sources of drinking water				
Improved	36 (10⋅3 %)	314 (89⋅7 %)	1	1
Unimproved	59 (9⋅5 %)	565 (90⋅5 %)	1⋅10 (0⋅71, 1⋅70)	0⋅96 (0⋅57, 1⋅61)

AOR, adjusted odds ratio; COR, crude odds ratio; CI, confidence interval; 1, reference.

* *P*-value <0⋅05 is considered significant.

** *P*-value <0⋅01 is considered significant.

## Discussion

In the present study, the prevalence of undernutrition and associated factors in the Amhara Region was assessed. The prevalence of stunting, underweight and wasting in the Amhara Region was 46⋅3, 28⋅4 and 9⋅8 %, respectively. In the present study, stunting and underweight are higher than the studies conducted in Ethiopia which were 38⋅3 and 23⋅3 %^(2)^, in Dale district 25⋅6 and 19 %^(12)^ and in Takusa district 36⋅5 and 19⋅5 %, respectively^(13)^. The prevalence of stunting and underweight in the present study is higher than the finding reported in Nairobi peri-urban slum 30⋅2 and 14⋅9 %, respectively^(14)^, but lower than the findings in Pakistan 489⋅2 and 39⋅5 %, respectively^(15)^. This could be because the households lack knowledge, attitude and practices (KAP) on how to feed their children and themselves^(16)^. Although the feeding practices for children in Ethiopia are generally poor, the complementary feeding practices are worse in the Amhara Region than in other parts of Ethiopia as a whole^(17)^. The prevalence of wasting in the present study is lower compared with the study conducted in Ethiopia 10⋅1 %^(2)^, in Haramaya district 10⋅7 %^(18)^, in Dale Woreda 14 %^(12)^, in Pakistan 10⋅7 %^(19)^ and 16⋅2 %^(15)^ and in Tanzania 24⋅7 %^(20)^. The prevalence reported in the present study is higher compared with the one reported in Nairobi peri-urban slum 4⋅5 %^(14)^. This divergence might be due to this region, it is a common practice to sell more nutritious food items such as legumes (beans, peas and chickpeas), sheep, goat, cattle, milk and milk products since they earn better income by selling these agricultural products rather than feeding these nutritious food items to their children^(21)^.

Among the factors considered in the present study, the age of child in months, birth weight, mothers’ educational level, sex of household head and sources of drinking water were associated with stunting. The log odds of stunting were higher among children in the age group of 25–47 and 48–59 months, respectively, as compared with the age group of 0–24 months. This finding is in line with the studies conducted in Ethiopia^(2)^, in Haramaya district^(18)^, in Pakistan^(19)^ and in Kilimanjaro Region, Tanzania^(20)^. Compared with children >4 kg birth weight at birth, the odds of stunting among children in the 2⋅5–4 kg birth weight were 0⋅01 times lower. The odds of stunting among children in the <2⋅5 kg birth weight at birth were 1⋅60 times higher compared with children >4 kg birth weight at birth. This finding is supported by a study conducted previously in Southern Nations, Nationalities and Peoples’ Region, Ethiopia^(22)^.

The risk of stunting among children whose mothers attended secondary education was 0⋅70 times less compared with children whose mothers never attended formal education. This finding is consistent with the study conducted in Bangladesh^(23)^ and in Pakistan^(19)^. Children born from educated women suffer less from stunting in children. Maternal education has been associated with nutrition outcomes among children in studies in various settings including Jamaica^(24)^. The risk of stunting among children whose father household head was 0⋅49 times less compared with children whose mother household headed. Children from households that used unimproved drinking water were 1⋅47 times more likely to be at risk of stunting than children from households that used improved water. This finding is supported by the result of similar studies conducted in Haramaya District, Eastern Ethiopia^(18)^.

The birth weight was associated with underweight (*P* < 0⋅05). The risk of being underweight was 1⋅36 times more likely among children that were aged 48–59 months than those aged 0–24 months. This finding is supported by the study conducted in Ethiopia^(2)^. The risk of being underweight for children whose mother attended primary and secondary education were 0⋅23 and 0⋅35 times lower than children whose mothers who did not attend formal education, respectively. This finding is supported by the study conducted in Ethiopia^(2)^ and in Pakistan^(19)^. The sources of discrepancy might be due to developing countries especially, maternal education has stronger child health and nutritional associations than paternal education^(25)^. It is also widely perceived that women, on average, wish to have fewer children than men and those mothers devote more resources to their children than fathers do^(26)^.

Children from a household with rich economic status were 0⋅09 times less likely to be underweight compared with children living in a household with poor household economic status. This finding is supported by the study conducted in Ethiopia^(2)^ and in Pakistan^(19)^. Children from rural areas were 1⋅16 times more likely to be underweight compared with children from urban areas. This finding is in agreement with the study conducted in Takusa district, Northwest Ethiopia^(13)^. While ample evidence documents that urban children generally have better nutritional status than their rural children. The environment, choices and opportunities of urbanites differ greatly from those of rural dwellers from employment conditions to social and family networks to access to health care and other services^(27)^. Female children were 1⋅06 times more likely to be underweighted as compared with male children. The present study is against the studies conducted in Ethiopia^(2)^, in Pakistan^(15)^, in Bule Hora district, South Ethiopia^(28)^ and in Dale Woreda, southern Ethiopia^(12)^. Children who were born with a <2⋅5 kg birth weight were 1⋅80 times more likely to be underweighted than children born >4 kg birth weight, and children who had born with 2⋅5–4 kg birth weight were 1⋅56 times more likely to be underweight than children born >4 kg birth weight. This finding is in agreement with the study conducted in Ethiopia^(2)^ and in Dale Woreda, southern Ethiopia^(12)^.

Results of the multivariable binary logistic regression model showed that the type of toilet facility and sex of household head were significantly associated with wasting. Children living in a household with improved toilet type were 0⋅48 less likely to be wasting compared with children living in a household with unimproved toilet type. This finding is in agreement with the finding in Bule Hora district, South Ethiopia^(28)^. Children from a male household head were 1⋅99 times higher compared with children from a female household head. Children of the rich household were 0⋅32 times less likely to be wasting compared with children living in a household with poor household economic status. This finding is supported by the study conducted in Ethiopia^(2)^ and in Pakistan^(19)^.

The risk of wasting was 1⋅08 and 1⋅52 times higher among children of 25–47 and 48–59 months than those of 0–24 months, respectively. This finding is supported by the studies conducted in Dale Woreda, southern Ethiopia^(12)^ and in Kilimanjaro Region, Tanzania^(20)^. The odds of being wasting of children from rural areas were 1⋅18 times higher compared with children from urban areas. This finding is consistent with the study conducted in Haramaya District, Eastern Ethiopia^(18)^. The study results suggest that lower urban malnutrition is due to a series of more favourable socioeconomic conditions, in turn leading to better-caring practices for children and their mothers^(29)^. The odds of wasting were 0⋅04 times lower among female children than male children. The present study is in line with the studies conducted in Bule Hora district, South Ethiopia^(28)^, in Dale Woreda, southern Ethiopia^(12)^, in Kilimanjaro Region, Tanzania^(20)^ and in Pakistan^(19)^. The odds of wasting was 1⋅17 times higher among children who lived in household members of >4 children who had lived in household members of 1–2. This finding is in line with the study conducted in Dale Woreda, southern Ethiopia^(12)^. Households with fewer children could be expected to be more capable than households with higher real income to provide their members with an adequate dietary intake^(30)^.

## Conclusion

The present study showed individual- and community-level factors determined childhood undernutrition in the Amhara Region children. Among the factors considered in the present study, the age of child in months, birth weight, mother educational level, sex of household head, sources of drinking water and the type of toilet facility were significantly associated with undernutrition in the Amhara Region. This finding would be directing the authors that undernutrition among under-five children was one of the public health problems in the Amhara Region. The potential risk factors should be considered to develop strategies for reducing undernutrition in the Amhara Region. Improving the living standards of children is important to get better health care, to enhance the child's nutritional status and to reduce child mortality. Finally, further researches on under-five children are recommended to investigate additional undernutrition factors.

### Limitations of the study

A limitation was the use of a cross-sectional study design which could only generate a hypothesis regarding the role of independent variables on the nutritional status of children but not their cause and effect relationships.
